# Dictionary Learning Based Scheme for Adversarial Defense in Continuous-Variable Quantum Key Distribution

**DOI:** 10.3390/e25030499

**Published:** 2023-03-14

**Authors:** Shimiao Li, Pengzhi Yin, Zehao Zhou, Jianheng Tang, Duan Huang, Ling Zhang

**Affiliations:** 1 School of Physics and Electronics, Central South University, Changsha 410017, China; 2 School of Automation, Central South University, Changsha 410017, China; 3 School of Computer Science, Central South University, Changsha 410017, China

**Keywords:** CVQKD, adversarial attack, sparse defense, locality preserving projects

## Abstract

There exist various attack strategies in continuous-variable quantum key distribution (CVQKD) system in practice. Due to the powerful information processing ability of neural networks, they are applied to the detection and classification of attack strategies in CVQKD systems. However, neural networks are vulnerable to adversarial attacks, resulting in the CVQKD system using neural networks also having security risks. To solve this problem, we propose a defense scheme for the CVQKD system. We first perform low-rank dimensionality reduction on the CVQKD system data through regularized self-representation-locality preserving projects (RSR-LPP) to filter out some adversarial disturbances, and then perform sparse coding reconstruction through dictionary learning to add data details and filter residual adversarial disturbances. We test the proposed defense algorithm in the CVQKD system. The results indicate that our proposed scheme has a good monitoring and alarm effect on CVQKD adversarial disturbances and has a better effect than other compared defense algorithms.

## 1. Introduction

Quantum key distribution (QKD) [1] enables Alice and Bob, two distant parties, to share security keys through quantum channels. In particular, although there is a possibility of being eavesdropped by Eve in the quantum channel, the quantum uncertainty principle [2,3] and quantum no-cloning theorem [4] theoretically guarantee the unconditional security of the QKD system. At present, according to the methods of QKD implementation, it can be divided into discrete–variable quantum key distribution (DVQKD) and continuous–variable quantum key distribution (CVQKD) [5]. Among them, CVQKD system, using mature coherent detection technology to replace the single-photon detection technology, which is susceptible to dark count noise, can be commendably compatible with the classical optical communication system. In addition, the CVQKD system also has the advantages of high key rate and easy preparation of signal carrier [6,7]. Therefore, it is important to study the CVQKD system for the practical application and promotion of quantum cryptography.

CVQKD based on Gaussian-modulated coherent states (GMCS) is a distinguished scheme [8]. In recent years, many researchers have realized GMCS CVQKD in different scenarios. At the same time, in the case of infinite and finite code length, the security of GMCS CVQKD system under collective attack and coherent attack has also been proved [9,10]. However, there are some vulnerabilities in the actual GMCS CVQKD system that do not meet the security proof assumptions. Eve can exploit these loopholes to steal key information through some attack strategies, such as wavelength attacks, homodyne-detector-blinding attacks, Trojan horse attacks, calibration attacks, local oscillator (LO) intensity attacks, and saturation attacks [11,12,13,14,15,16]. Quantum hacking attacks seriously undermine the system’s practical application security. In order to defend against such attacks, researchers have proposed to add suitable real-time monitoring modules or measurement devices, which will discard the entire key data once an attack is detected, leading to a waste of time and resources [17,18,19]. More importantly, we cannot know what kind of attack Eve will launch in advance. To solve this problem, Mao et al. [20] and Luo et al. [21] proposed to classify attacks with deep learning and perform corresponding defense strategies, according to the classification result.

However, neural networks are often vulnerable to subtle perturbations, namely adversarial attacks, such as fast gradient sign method (FGSM), projected gradient descent (PGD), Deepfool, antagonistic network for generating rogue images (ANGRI) [22,23,24,25], etc., leading the neural networks to predict incorrectly. What is worse is that recent studies have shown that adversarial attacks can be applied to the real world by those possessing ulterior motivates, which pose a serious threat to areas requiring high information security, for example, autonomous driving [26]. Similarly, for the CVQKD system using neural networks for attack detection and classification, adversarial attacks can also affect the CVQKD system, which is proved by our simulation experiments [27].

So far, many researchers have proposed defense strategies against adversarial attacks. For example, Li et al. [28] applied adversarial samples generated by PGD for adversarial training. Yuan et al. [29] trained the target model using adversarial examples to increase the robustness of the model. Su et al. [30] proposed to transform the images with adversarial disturbances into benign images through the probabilistic model of Pixel CNN. According to the characteristics of quantum key transmission protocol in CVQKD system, we propose an adversarial attack detector. It detects adversarial attacks through low-rank dimensionality reduction and sparse reconstruction, which can detect and alarm in time when there is an adversarial disturbance in the testing sample.

In our experiment, we first perform low-rank dimensionality reduction on clean datasets through regularized self-representation-locality preserving projects (RSR-LPP), using the output data for offline dictionary learning. For the online defense stage, we also introduce low-rank dimensionality reduction on the datasets through RSR-LPP to filter some disturbances. After that, in order to add the details of the datasets lost in the dimensionality reduction process and monitor the remaining disturbances, we reconstruct the datasets by multi-scale sparse coding, in which the dictionary used for reconstruction is learned in the offline stage. If the datasets contain adversarial disturbances, the reconstruction error will be too large to cause an alarm, realizing the monitoring and filtering of adversarial samples.

This article is structured as follows. Firstly, in Section 2, we introduce the datasets preparation, the process of neural networks construction and the commonly used adversarial attack algorithms. In Section 3, we propose a defense algorithm based on low-rank dimensionality reduction and sparse reconstruction. Then in Section 4, we simulate the proposed defense algorithm in CVQKD system and make a comparison between the experimental results and other defense algorithms, proving the efficiency of our proposed defense algorithm. Finally, we make a brief summary in Section 5.

## 2. Preliminaries

### 2.1. Datasets and Parameter Settings

The quantum state of encoding information in CVQKD is infinite dimensional and continuous in Hilbert space, corresponding to many protocols. According to the different ways of coding modulation, the protocols can be divided into Gaussian modulation and discrete modulation. According to the different light sources used in the system, they can be divided into squeezed state, coherent state, and entangled state. From the perspective of practical application, the preparation of squeezed state and entangled state is relatively difficult, so the GMCS CVQKD is the most widely used and accepted protocol [31]. The protocol uses the easily prepared coherent state as the light source and uses a relatively low cost balanced zero beat detector as the detection end, which can break 3 dB theory of the channel [32].

**Figure 1:**
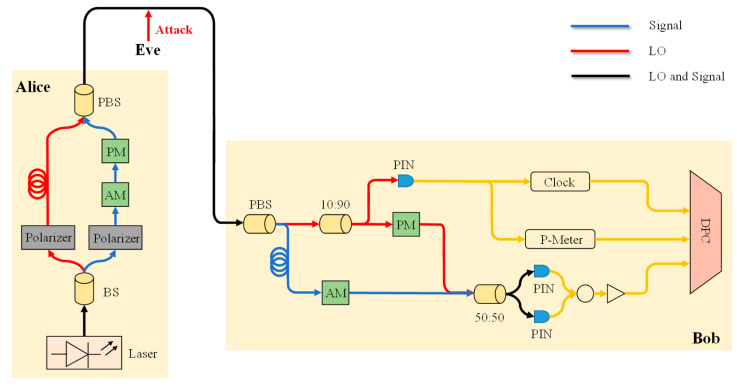
The schematic diagram of the whole experimental device for obtaining the training data of neural networks. AM: amplitude modulator. PM: phase modulator. PBS: polarization beam splitter. PIN: PIN photodiode. CLOCK: clock circuit used to generate clock signal for measurement. P-METER: power meter. DPC: data processing center.

In the GMCS CVQKD protocol, as shown in Figure 1, Alice first uses a laser to generate an optical pulse, and then uses a random number generator to generate a Gaussian random number pair ($X_{t},P_{t}$). After that, Alice modulates the optical pulse through an amplitude modulator and a phase modulator to obtain a series of coherent states $|X_{t}+P_{t}\rangle$. $N_{0}$ represents the variance of shot noise, the orthogonal values $X_{t}$ and $P_{t}$ obey the bivariate Gaussian distribution, the mean is zero, and variance is $V_{t}N_{0}$. Then, Alice sends the coherent quantum signal to Bob through a quantum channel. Since quantum channels are easily attacked by Eve, quantum channels are generally considered unsafe.

In Bob’s path, a homodyne detection is applied to the quantum signal, and adversarial attacks can be added into LO and signal, as shown in Figure 1. Bob randomly chooses to switch the measurement base (0, π/2) to measure the received quantum state with balanced zero beat. Firstly, Bob uses a polarization beam splitter (PBS) to demultiplex the signal pulse and the local oscillator (LO) pulse. Then, in the signal path, Bob uses an amplitude modulator (AM) to randomly reduce 10% of the signal pulse for scattering noise estimation, and retains the rest signal pulse without change. In the LO path, the LO pulse is split at a ratio of 10:90. The smaller part of the LO pulse is applied to measure the LO power and generate a clock, and the rest is used for homodyne detection. After that, the measurement results can be sent to the data processing center (DPC) to sample and detect attacks.

Through the process, we can obtain two series of related data $x=\left\{\;x_{1},\;x_{2},\ldots ,\;x_{N}\;\right\}$ and $y=\left\{\;y_{1},\;y_{2},\ldots ,\;y_{N}\;\right\}$, where x is the quadrature value generated by Alice and y is the quadrature value measured by Bob. From x and y, we can retrieve multiple features. Different attack strategies have an impact on different features. We mainly study five attack strategies, including calibration attack, LO intensity attack, saturation attack, hybrid1 attack and hybrid2 attack. We notice that these five attack strategies mainly have different effects on $\bar{y},\;V_{y},\;I_{LO},N_{0}$, where $\bar{y}$ represents the mean, $V_{y}$ represents the variance of Bob’s measurement, $I_{LO}$ is the intensity of local oscillator light and $N_{0}$ indicates the variance of shot noise. The values of the feature vector are different under different types of CVQKD attacks since different attacks act on different features and change their values in different ways. Take the calibration attack that Eve may manipulate into attacking the LO signal as an example. The calibration attack is specifically aimed at the system that takes the LO as the clock signal and monitors its strength in real time. In this attack, the variance of the shot noise $N_{0}$ is overestimated after the clock signal is delayed, resulting in a security vulnerability. Calibration attacks can be detected by monitoring the increase of $N_{0}$. Therefore, we can judge which attack Eve has applied according to the change of the features. Therefore, we construct a classification neural network and combine these four features to form a feature vector $\vec{u}=\left\{\;\bar{y},\;V_{y},\;I_{LO},N_{0}\;\right\}$, and then construct a function f:u → v through deep learning. This is constructed so that we can obtain an output vector $\vec{v}$ from an input vector $\vec{u}$, and tell the type of attack Eve has applied according to the output vector $\vec{v}$.

Our data set preparation steps used for neural network training and testing are as follows: First, for each attack strategies and normal condition, we generate the original data sets of N pulses in chronological order. Then, the original data sets are divided into M blocks. After that, we calculate the data of each block to obtain four features, which are combined into the feature vector $\vec{u}=\left\{\;\bar{y},\;V_{y},\;I_{LO},N_{0}\;\right\}$. Finally, in order to obtain more scientific and reasonable training sets and test sets, we put all the data sets together, disrupt the order and select 70% of them as the training sets. The remaining 30% are the test sets. Here, all the work of preparing the training sets and the test sets has been completed. 

### 2.2. The Establishment of Neural Network and Training Results

The adversarial samples are generated by multiple iterations or queries on the neural network, which means the neural network is not only the object of adversarial sample attacks, but also the basis for the generation of adversarial samples. The commonly used neural networks are Artificial Neural Network (ANN), Convolution Neural Network (CNN), Recurrent Neural Network (RNN), and Deep Neural Network (DNN) [33,34,35]. We mainly introduce ANN and CNN, and build corresponding models for experiments.

**Figure 2:**
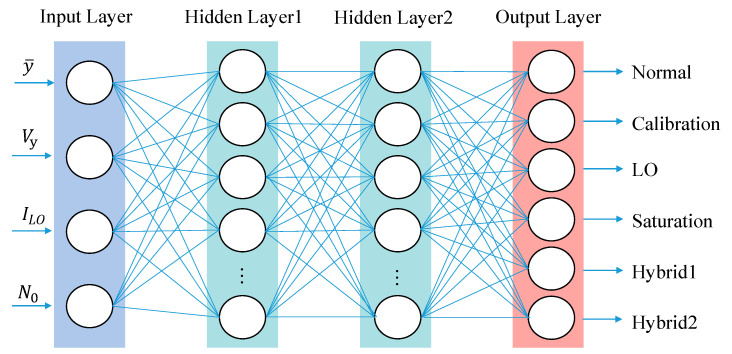
The Artificial Neural Network (ANN) we constructed.

ANN is a kind of machine learning model, which mainly imitates the structure and function of biological neural network. The ANN network structure used in this experiment is shown in Figure 2. In a neural network, the number of neurons in the input layer is generally determined by the characteristics of the input data, and the number of neurons in the output layer is usually targeted to the problems. In this experiment, the feature vector extracted from CVQKD sampling data contains four elements, and we need the data to be divided into six categories through ANN, which are normal data and five CVQKD system attack strategies. So, the input layer and output layer are set to contain four and six neurons, respectively. The settings of the input layer and the output layer are relatively fixed, while the settings of the hidden layers are flexible. The number of layers and the number of neurons can be set according to actual needs, so the setting of the hidden layer greatly affects the role of the neural network.

**Figure 3:**
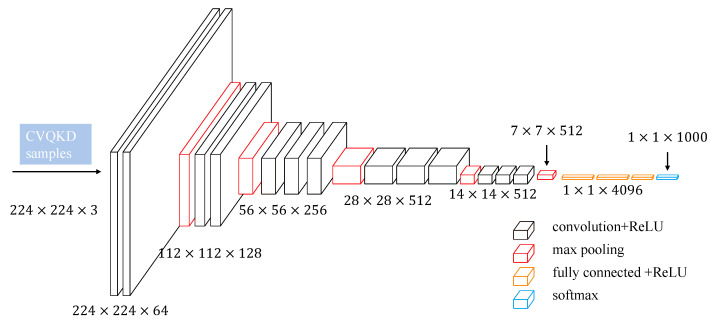
The Convolution Neural Network (CNN) we constructed.

ANN is a typical neural network model which is simple and easy to build. The neurons of adjacent two layers are connected in pairs, and the information of each input dimension will affect the neurons in the back layer. However, because all layers are fully connected, the weights and bias parameters in the network will increase exponentially with the increase of the number of layers, which makes the neural network training process more difficult and the convergence rate slow. In view of the above problems, the researchers conducted in-depth research and proposed CNN. In this paper, in order to verify that the adversarial attack can effectively attack a variety of neural networks applied to the CVQKD system, a CNN model, with the structure shown in Figure 3, is constructed. We extract the features of the sampled data of the CVQKD system and simply modify its form to meet the requirements of CNN input layer parameters. The CNN network structure used in this experiment includes four core modules, namely convolution layer, pooling layer, fully connected layer, and Softmax layer. 

**Figure 4:**
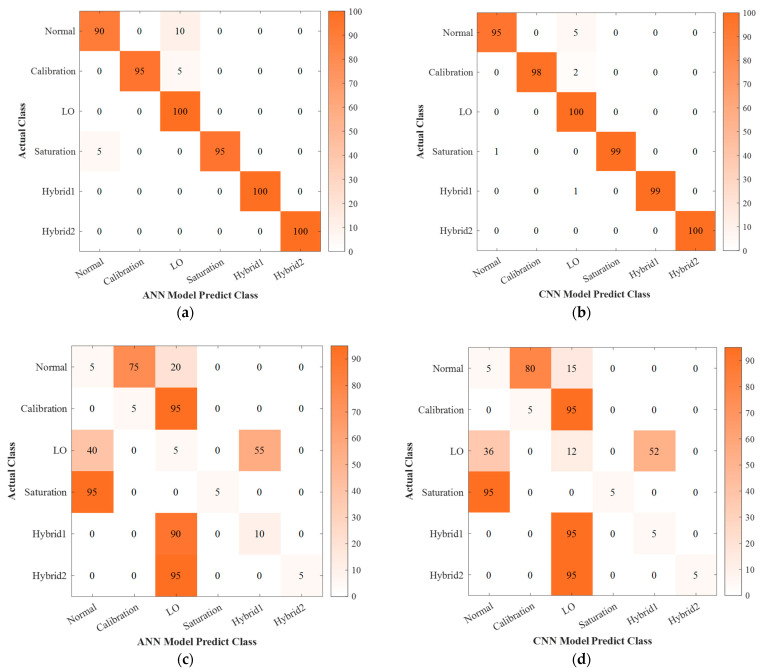
The confusion matrix diagram of ANN and CNN for continuous-variable quantum key distribution (CVQKD) attack detection before and after being attacked. (**a**,**b**) are the ANN and CNN classification accuracy for CVQKD attacks, respectively. (**c**,**d**) show the ANN and CNN classification accuracy after adversarial perturbations added in the CVQKD system, respectively.

As our input data is relatively simple, we have made appropriate modifications to the parameters of the neural networks, and it is proved that these modifications are effective through our experiments. As shown in Figure 4a,b, the deep color blocks are located on the diagonal, and the remaining blocks are approximately equal to 0, indicating that the neural networks constructed in our experiment have an excellent classification effect on the CVQKD attack strategies.

### 2.3. Adversarial Attack

Since the neural network is vulnerable to the threat of adversarial samples, the CVQKD system using neural network for attack detection and classification also has the possibility of being affected by adversarial samples. We perform the corresponding experiments, and the results are shown in Figure 4c,d in the form of confusion matrices. The color blocks with different shades are scattered in the pictures, indicating that adversarial attacks have an impact on the CVQKD system using neural networks for attack detection and classification. In order to find a defense strategy that has an effect on a variety of adversarial attacks, it is necessary to first learn about the commonly used adversarial attacks. At present, the commonly used adversarial attacks include Fast gradient sign method (FGSM), Deepfool, projected gradient descent (PGD), Carlini and Wagner attack (C&W), universal adversarial perturbation (UAP), and L-BFGS. We mainly introduce the first two attack methods.

- FGSM: Goodfellow et al. [36] proposed a simple fast gradient adversarial attack method. The main idea is to find the direction with the largest gradient change of the deep learning model, and add adversarial disturbances according to this direction, resulting in wrong classification of the model. This is a non-target attack method which is calculated as follows:

(1) $$x^{adv}=x+\epsilon \times sign\left(\nabla_{x}J\left(g\left(x\right),y_{ture}\right)\right)$$

In the formula, $g\left(x\right)$ is the recognition classification result of the target network. ϵ is the attack step size, which controls the strength of the attack, and $y_{ture}$ is the real category of x. However, in order to attack successfully, this attack will introduce a relatively large disturbance.

- Deepfool: Moosavi-Dezfool et al. [37] improved the generation strategy of adversarial samples and proposed an iterative method to calculate the minimum adversarial disturbance, and gradually pushed the samples within the legal sample boundary out of the boundary, resulting in wrong classification. They defined a new index in their paper to evaluate the classifier robustness. For a classifier $\hat{k}$, we define the distance between x and decision boundary as:

(2) $$∆\left(x;\hat{k}\right):=min_{r}\left|\left|r\right|\right|subject\;to\;\hat{k}\left(x+r\right)\neq \hat{k}\left(x\right)$$

and we define the robustness of the classifier $\hat{k}$ as:

(3) $$\rho_{adv}\left(\hat{k}\right)=E_{x}\frac{∆\left(x;\hat{k}\right)}{{\left|\left|x\right|\right|}_{2}}$$

where $E_{x}$ represents the expectation of input distribution. From the above two formulas, we can conclude that when we reduce the distance between x and the decision boundary, the robustness of the classifier $\hat{k}$ will be reduced. For a multi-classification task:

(4) $$\hat{k}\left(x\right)=\underset{k}{argmax}f_{k}\left(x\right)$$

where $f_{k}\left(x\right)$ represents a sub-classifier of the $k_{th}$ category. If we want to achieve the purpose of misclassification, we must ensure that at least one classifier scores higher than the target classifier. We defined the $k_{th}$ classification boundary as:

(5) $$F_{k}=\left\{x:f_{\hat{k}\left(x_{0}\right)}\left(x\right)-f_{k}\left(x\right)=0\right\}$$

As long as we find the shortest path from x to the decision boundary through multiple iterations, we can find the minimum disturbance that makes the classifier $\hat{k}$ misclassified. Compared with the FGSM attack method, the Deepfool attack method will introduce smaller perturbations and obtain higher attack success rates.

We summarize all the above attack methods in Table 1.

## 3. Defense Strategy

According to different defense ideas, we divide the existing adversarial defense algorithms into three types.

The first type consists of adding adversarial disturbances to a part of the training samples of the neural network and enhancing the robustness by making the model learn the characteristics of the disturbance. Zheng et al. [38] put the images without adversarial disturbances and the images with Gaussian noise into the classification neural network in pairs for adversarial training. Metzen et al. [39] trained the adversarial perturbation detector by labeling the original images as positive samples and the disturbed images as negative samples. Adversarial training can improve the robustness of neural network to adversarial disturbance samples, but the effect is not significant, and additional computing resources are required to generate adversarial disturbance samples and train the neural network.

The second type consists of modifying the activation function of the neural network and changing or adding the network structure to smooth the model and enhance the stability of the model. Zantedeschi et al. [40] modified the Relu function of the original network to a fixed upper bound Relu function to limit the layer-by-layer amplification of the disturbance. Ross et al. [41] added a gradient regularization term to the original cost function to smooth its gradient in the input space. Samangouei et al. [42] reconstructed the input image by training the generator of GAN. Such methods consume less additional computing resources, but they are not robust enough when they are confronted with gray-box attacks or white-box attacks.

The third type consists of preprocessing the images before image classification, so as to achieve the purpose of weakening the adversarial disturbances. Xu et al. [43] compressed and reconstructed the sub-network, by first compressing the image to 12 bits, and then reconstructed the image. Such methods are generally not affected by attack algorithms, classification models or data sets, and have good robustness in the face of gray-box attacks or white-box attacks. At the same time, because their end-to-end gradient is not easy to calculate, they also have good defensiveness against secondary attacks based on gradient. The proposed defense algorithm belongs to the third type.

**Figure 5:**
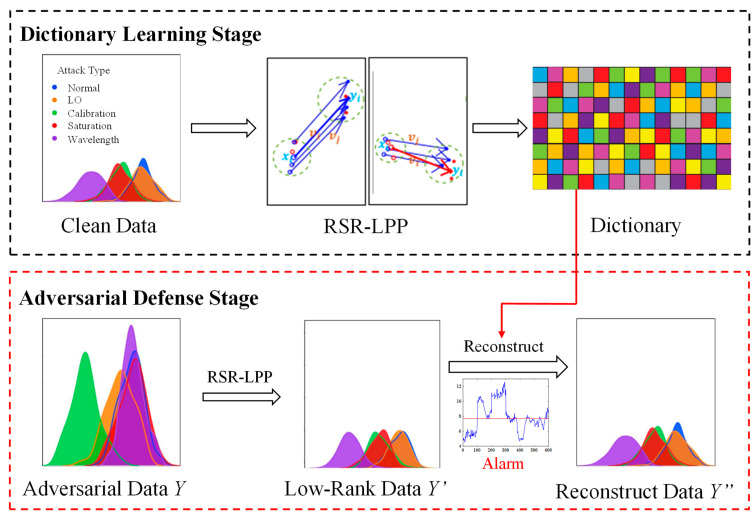
The schematic diagram of the proposed algorithm.

We first use regularized self-representation-locality preserving projects (RSR-LPP) to obtain the low-rank representation of the data, so as to filter the non-low-rank adversarial perturbations different from the natural data. The idea to map complex input data into low-dimensional feature spaces to efficiently separate them [44] inspires us to propose a low-rank dimensionality reduction on clean datasets to defend against the adversarial samples. However, the low-rank data still has residual adversarial disturbances, and the process of low-rank dimensionality reduction will also lose some details of the original images. Therefore, the proposed algorithm performs multi-scale sparse coding reconstruction on the projected low-rank data. The dictionary used in the reconstruction process is learned from the natural images, which ensures that the dictionary atoms are undisturbed and can combine the details of the natural images. The complete process of our proposed algorithm is shown in Figure 5. The upper half is the process of the dictionary learning in the offline stage of the algorithm. We first use the undisturbed natural data in the training set to construct the regularized projection set, and then use the low-rank data to learn the corresponding level of dictionary. The lower half of Figure 5 is the image preprocessing step in the online stage of the algorithm and includes the following steps: obtaining the low rank representation of the disturbed images through RSR-LPP; sparse coding reconstruction of the low-rank representation; and finally, the reconstructed data is obtained. In the reconstruction process, a monitoring mechanism is introduced to alarm the adversarial disturbances.

### 3.1. Regularized Self-Representation-Locality Preserving Projects (RSR-LPP)

The data dimension reduction model proposed in this paper is based on the two ideas of feature self-expression and data regularization. Among them, each feature in the regularized self-representation (RSR) model is linearly represented by other important features, thus forming a feature representation term to preserve the local geometric structure of the data. Assuming that there is a sample matrix $Z=\left[z_{1},z_{2},\ldots ,z_{d}\right]\in R^{n\times d}$, where n and d represent the number of samples and the number of features of the sample matrix, the feature self-expression model can be defined as:

(6) $$z_{i}\approx \sum_{j=1}^{d}z_{j}w_{ji},i=1,2...d$$

where $w_{ji}$ represents the weight between features $z_{i}$ and $z_{j}$.

Locality Preserving Projects (LPPs) optimally preserve the neighborhood structure of data by obtaining linear projections. It makes the samples maintain structural relationship after nonlinear dimensionality reduction. Assuming that W is a projection matrix that projects the sample data into a low-dimensional subspace, the optimal solution can be obtained by optimizing the following objective function:

(7) $$\underset{W}{min}\sum_{i,j=1}^{n}||W^{T}z_{i}-W^{T}z_{j}{||}^{2}s_{ij}$$

where W is the projection matrix and S is the similarity matrix. The elements are defined as follows:

(8) $$s_{ij}=\left\{\begin{array}{ll} \exp(-\frac{||z_{i}-z_{j}{||}_{2}}{2\sigma^{2}}) & ,z_{i}\;and\;z_{j}\;are\;neighbors\\ 0 & ,else \end{array}\right.$$

where σ is the width parameter.

By locally preserving the projection of the perturbed data, we obtain a reduced-dimensional data model. The main information is retained, and the adversarial disturbances are reduced to a certain degree, thereby improving the accuracy of classification.

### 3.2. Reconstruction of Sparse Coding

A part of data information will be lost in the process of local preserving projection of data, including adversarial disturbances and original data information. When using RSR-LPP to reduce the dimension of data, it is impossible to ensure that the adversarial disturbances are greatly weakened and the image information is retained, which will affect the accuracy of classification. To solve this problem, we use sparse coding to reconstruct RSR-LPP data, so as to add the detailed information of the original data and filter the residual adversarial disturbances. The dictionary used in sparse coding reconstruction is trained with normal data. The dictionary contains the original features of the data, and the detailed information of the original data can be reconstructed by the linear combination of atoms. For the sake of matching the data features of CVQKD system, we propose a dictionary learning algorithm.

The dictionary learning algorithm is data adaptive. Its main idea is to learn a group of atoms from the original data and take use of a dictionary, which is composed of the sparse linear atoms to approach the original data infinitely. Let $Y=\left[Y_{1},Y_{2},\ldots ,Y_{M}\right]\in R^{m\times M}$ be a data matrix, where M is the number of samples and m is the dimension of the original data. In order to obtain a suitable dictionary D for sparse linear reconstruction of original data, we need to solve the following formula:

(9) $$\langle D,X\rangle =\arg\underset{D,X}{\min}||Y-DX|{|}_{2}^{2},\;s.t.\forall_{i},||x_{i}|{|}_{0}\leq T$$

where $D=\left[d_{1},d_{2},\ldots ,d_{K}\right]\in R^{m\times K}$ represents a dictionary which contains K atoms, $X=\left[x_{1},x_{2},\ldots ,x_{K}\right]\in R^{K\times m}$ represents the sparse coding of the dictionary D, and $|\left|Y-DX\right|{|}_{2}^{2}$ represents the reconstruction error. $|\left|x\right|{|}_{0}$ is the $L_{0}$ norm of vector x, and T indicates the sparse constraint. In order to get a reasonable result of the above formula, we often use method of optimal directions (MOD) or K-SVD algorithms. 

After obtaining the dictionary D, clean data samples can be obtained by sparse reconstruction to solve the following optimization problems:

(10) $$\underset{a}{min}||Y^{\prime }-Y^{\prime \prime }|{|}_{F}^{2}=\underset{a}{min}||Y^{\prime }-Da|{|}_{F}^{2},s.t.||a|{|}_{0}\leq T^{\prime }$$

where a is the column vector of sparse coding X, $Y^{\prime }$ is the low-rank data reduced by RSR-LPP, and $Y^{\prime \prime }$ is its reconstruction. When the above formula T and $T^{\prime }$ are consistent, the dictionary’s detailed combination reconstruction $Y^{\prime \prime }$ will have the detail information closest to the natural images. Moreover, the dictionary learning method also has the function of monitoring, which can alarm against adversarial disturbances, realizing the alarm and weakening adversarial samples. When the reconstruction error exceeds the control limit, the test data will be considered abnormal, so the scientific calculation of control limit determines the monitoring effect. We use nonparametric kernel density estimation (KDE) [45] to obtain the control limit of the process in the target domain:

(11) $${\tilde{f}}_{h}\left(e\right)=\frac{1}{n}\sum_{i=1}^{n}K_{h}\left(e-e_{i}\right)=\frac{1}{nh}\sum_{i=1}^{n}K_{h}\left(\frac{e-e_{i}}{h}\right)$$

where ${\tilde{f}}_{h}\left(e\right)$ represents the density function, h represents the bandwidth, $K\left(•\right)$ indicates a nonnegative kernel function, and $\left(e_{1},e_{2},\ldots ,e_{N_{T}}\right)$ is the reconstruction error of the data trained in the target domain. Given the confidence level, we can obtain the control limit drr by the density function ${\tilde{f}}_{h}\left(e\right)$.

## 4. Performance

**Figure 6:**
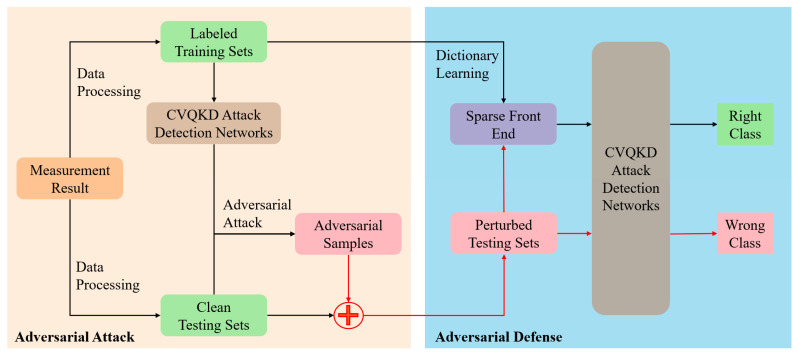
The framework of adversarial defense based on sparse front-end and its preparation process.

The CVQKD simulation and the dataset generation are programmed in Matlab R2019b, and then the attack and defense training models are programmed in Python 3.7 with PyTorch. As shown in Figure 6, for the sake of verifying the actual effect of adversarial attacks and adversarial defenses, we create a batch of CVQKD data as attack object. We select 4000 sets of data as the training set, of which 3000 sets of data contain attacks on the CVQKD system, namely Calibration, L0, Saturation, Hybrid1, and Hybrid2. Each attack strategy corresponds to 600 sets of data, and the remaining 1000 sets are normal data. Six dictionaries are learned from these data to realize the alarm and filtering of adversarial disturbances, named $D_{1}$, $D_{2}$, …, $D_{6}$. Then, we conduct adversarial attacks on the input data. In this paper, Deepfool is used for testing, Δ is 0.04, and for each attack strategy, we add perturbations to its corresponding last 200 sets of data. We select another 3000 sets of data as the test set, of which 2500 sets of data contain attacks on the CVQKD system. Each attack strategy corresponds to 500 sets of data, and the remaining 500 sets are normal data. Among each 500 sets of data, the first 300 sets contained no adversarial disturbances, and the last 200 sets contained adversarial disturbances. We used ANN as the CVQKD attack detection classification network. 

The evaluation indexes of our proposed model are as follows:

(12) $$FAR=\frac{FP}{FP+TN},FDR=\frac{TP}{FP+TN}$$

where False Accept Rate (FAR) represents the probability that false samples are judged as normal samples by the model, which may lead to security risks in the system. False Discovery Rate (FDR) represents the probability that false samples are found by the model, which reflects the error detection ability of the model. False positive (FP) refers to the number of cases that do not belong to this category but are determined to be in this category. True positive (TP) refers to the number of cases that belong to this category and are judged as such. True negative (TN) refers to the number of cases that do not belong to the category and are not determined to belong to the category.

**Figure 7:**
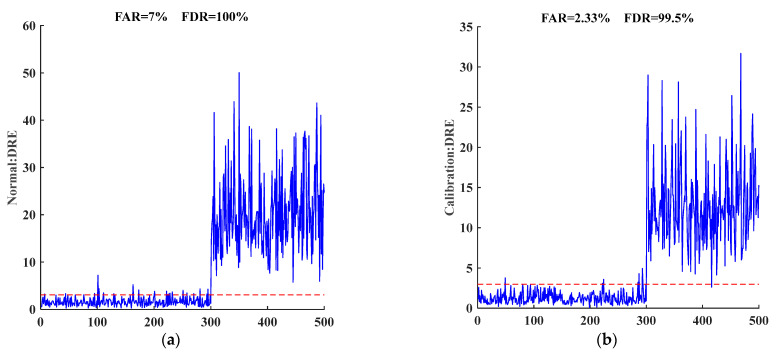

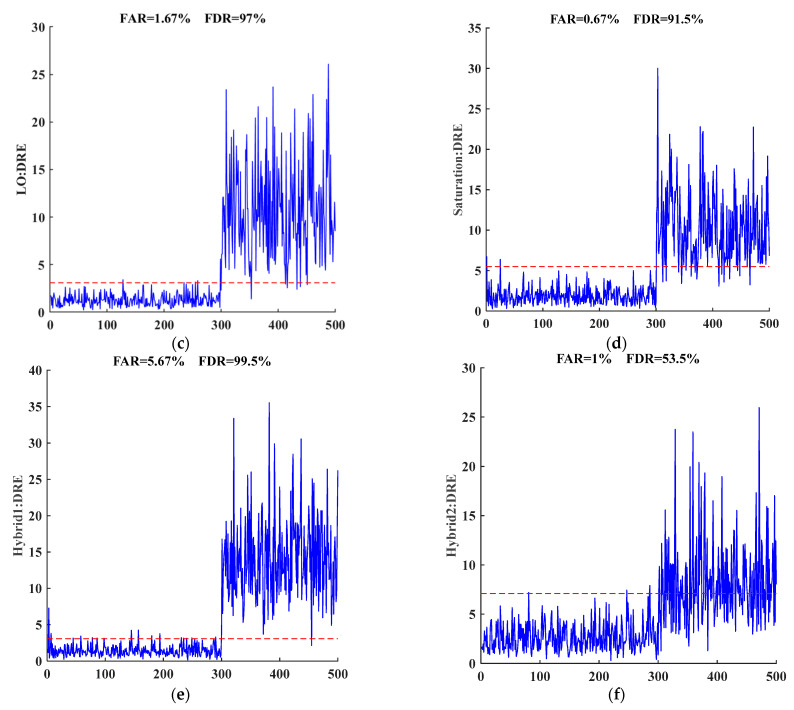
The defense effect diagram of different attack strategies for the CVQKD system. The blue line represents the reconstruction error and the red line represents the control limit. When the blue line is higher than the red line, it is regarded that the data contains adversarial disturbances.

Figure 7a–f represent the dictionary learning monitoring and alarm effects of six types of data with or without adversarial disturbance. The first 300 sets of data were not added to the adversarial perturbation, and the last 200 sets of data were added to the Deepfool perturbation. Because of its powerful data representation ability, dictionary learning can learn CVQKD data structure well and generate a complete dictionary. It can be seen from the first 300 data of each picture that the reconstruction error is within the reliable range, and the FAR is small, with a maximum of 7% and an average of 3.06%, which reflects that the dictionary learning method has a low false alarm rate. At the same time, the FDR of the first five data after adding Deepfool perturbation is very high, more than 91.5 %, indicating that dictionary learning is sensitive to adversarial perturbation and can distinguish adversarial perturbation well. In the case of Figure 7f, FDR is only 53.5%. This is because Hybrid2 is a mixture of multiple attack strategies, its noise is large, and it is easily confused into the other attack strategy under Deepfool attack. This shows that the dictionary learning method has a good monitoring effect on the adversarial perturbation of single attack data, and the effect of adversarial perturbation under some mixed attacks is slightly worse. It is worth noting that the performance of common adversarial defense strategies will be reduced in this case, which is the future research direction.

**Figure 8:**
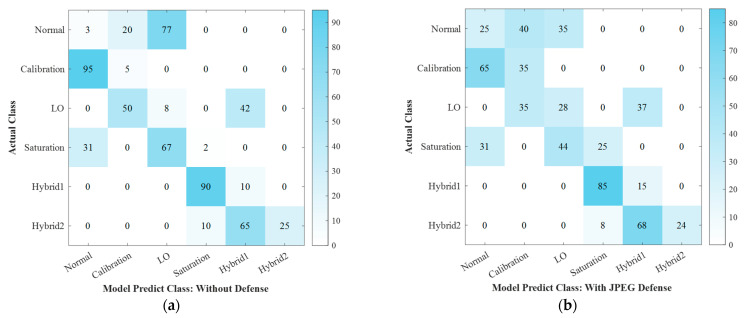

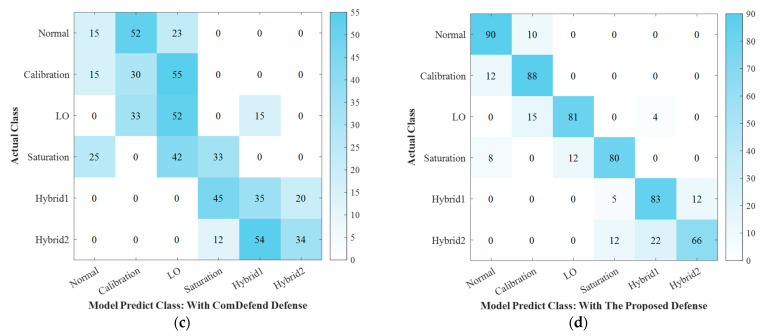
Model classification effect before and after introducing different adversarial defense algorithms.

In addition, we conducted a comparative experiment on the classification effect of the ANN model before and after adding adversarial defense, where the Deepfool Δ is 0.4. Figure 8a shows the model classification effect without introducing the adversarial defense algorithm, and Figure 8b–d is the classification effect of the model with different adversarial defense algorithms, which are JPEG, ComDefend, and the defense model proposed in this paper. The comparison algorithm uses the open-source code and the best parameter settings provided by the corresponding literature for experiments. The comparison shows that the defense strategy proposed in this paper has a good effect in the CVQKD system. In addition to Hybrid2’s adversarial disturbances filtering effect being slightly worse, the remaining classification accuracy is better than the comparison methods. The average accuracy comparison is shown in Table 2. It can be seen that the method proposed in this paper is a very effective CVQKD system adversarial defense strategy, showing great advantages in filtering adversarial attacks.

Since adversarial attacks and defenses originate from the image classification field, in order to more intuitively prove the effectiveness and versatility of the proposed defense method, we also carried out verification experiments on the public image dataset. This is because the data transmitted by CVQKD channel can be simulated to matrix data, in which the principle of training detection model is the same as that of image data. Most of the anti-attack methods against images may be applied to CVQKD data, so it is important to illustrate the efficiency of the proposed method in the public image data test. We select pre-trained EfficientNet-B7 and ResNet152 as the classification models, whose Top-1 classification accuracies on the ImageNet test set are 85.0% and 77.8%, respectively. The dictionary learning training set uses 1000 images randomly selected from the ImageNet dataset. For the sake of verifying the robustness of our proposed algorithm, two adversarial attack algorithms, FGSM and Deepfool, are used to attack the deep learning model, respectively. Each attack algorithm generates four test sets with different levels of disturbance size (Δ = 0.01, 0.02, 0.03, 0.04). A total of eight test sets are generated, and the attack images are all from the ImageNet dataset. Moreover, for the sake of verifying the defense effect of the proposed algorithm, four other defense algorithms are selected under the same conditions as the above test environment for comparative experiments, which are JPEG compression (JPEG), total variance minimization (TVM), pixel deflection and wavelet blurring (PDWD), and Codefined. The first three algorithms belong to the traditional adversarial defense processing algorithms, and the latter belongs to the adversarial defense algorithm based on convolutional neural network. The above comparison algorithms are tested with the open-source code and the best parameter settings provided by the corresponding literature. All five algorithms conduct adversarial defense experiments under two attack modes: black-box attack and gray-box attack. The black-box attack is an attack carried out by the attacker without any information, such as the defense strategy, classification model type, and parameters of the defender. In order to simulate the black-box attack scenario, the defender is set to use ResNet152 for classification, and the attacker attacks the EfficientNet-B7 model without knowing any information of the defender. Grey-box attack is an attack in which the attacker has a target in the case of known defense strategy or classification model type and parameters. In order to simulate the gray-box attack scenario, the defender uses ResNet152 for classification, and the attacker knows this information and attacks the ResNet152 model to generate disturbance data. In the eight test sets, the Top-1 classification accuracy results of the five defense algorithms in the black-box attack experiment and the gray-box attack experiment are shown in Table 3. Among them, the left side of ‘/’ is the defense black-box attack accuracy and the right side is the defense gray-box attack accuracy.

It can be seen from Table 3 that the proposed algorithm performs better than the comparison algorithm on seven test sets in the experiment of defending black-box attack, and the proposed algorithm achieved a lead on eight test sets in the gray-box attack experiment. In terms of the overall average classification accuracy, the proposed algorithm is generally superior to the other four comparison algorithms, leading the second algorithm by 3.1/3.6 percentage points. Among the four comparison algorithms, JPEG and TVM are similar to the proposed algorithm, because they do not rely on the classification model or any attacker information in data processing. However, the basic principle of these dealing with adversarial perturbations changes the data information by a large margin, which changes the adversarial perturbation information at the same time to make it invalid. This change will lose the details of the original data and reduce classification accuracy of processed images. In contrast, the proposed algorithm performs sparse coding reconstruction on CVQKD system data that filters adversarial disturbances, which effectively solves this problem.

Table 4 shows the ablation experimental results of the proposed algorithm. In the table, ‘LPP’ indicates that the data is only processed by LPP, and ‘RSR-LPP’ indicates that the data is processed by RSR first and then LPP. The display form of the data is the same as Table 3. From the table, we can find that the RSR-LPP method proposed in this paper has a significant effect on improving the classification accuracy of CVQKD data.

## 5. Conclusions

In this paper, we propose an algorithm to defend against adversarial attacks in the CVQKD system. We first prove the possibility of adversarial attacks in CVQKD system through experiments, and then introduce the dictionary learning based scheme, which realizes the filtering of adversarial disturbances through low-rank dimensionality reduction and sparse coding reconstruction. We apply the proposed algorithm to the CVQKD system, proving that our proposed algorithm has a great defense effect on CVQKD systems and is more efficient than the compared algorithms. To verify the versatility of the proposed algorithm, we conduct experiments on another dataset, filtering images with different types and sizes of adversarial attacks using the proposed algorithm. It is worth noting that for the adversarial perturbation under mixed attacks, the current adversarial defense effect is generally poor, which is the direction of our research in the future. The dictionary learning based defense scheme can protect AI assisted physical instruments against adversarial attacks, improving their security.

## Figures and Tables

**Table 1:** Summary of the characteristics of various adversarial attacks.

Method	Algorithm	Perturbation Norm	Victim Information
FGSM	attacks based on gradient	$l_{2}\;,\;l_{\infty }$	white-box
Deepfool	attacks based on gradient	$l_{2}$	white-box
PGD	attacks based on gradient	$l_{\infty }$	white-box
L-BFGS	attacks based on optimization	$l_{2}$	white-box
C&W	attacks based on optimization	$l_{p}$	white-box
UAP	attacks based on optimization	$l_{2}\;,\;l_{\infty }$	black-box

**Table 2:** The average classification accuracy before and after introducing different adversarial defense algorithms.

Method	Average Accuracy
With no defense	0.8%
JPEG	25.3%
ComDefend	33.1%
The proposed method	80%

**Table 3:** Top-1 classification accuracy of each defense algorithm. The bold are the best results among the five defense algorithms.

Attack Algorithm	Δ	Proposed Algorithm	JPEG	TVM	PDWD	ComDefend
FGSM	0.01	96.4/94.0	80.8/88.9	92.1/85.8	88.0/86.5	88.5/87.3
	0.02	94.2/92.3	87.0/88.0	91.6/84.0	86.1/84.0	87.0/85.1
	0.03	91.1/86.0	88.6/83.4	90.9/83.5	82.7/80.1	85.3/83.3
	0.04	89.2/80.9	85.4/78.9	89.7/79.5	81.1/80.4	86.4/78.7
	Average	92.7/88.3	85.5/84.8	91.1/83.2	84.5/82.8	86.8/83.6
Deepfool	0.01	89.2/86.3	85.8/81.2	83.0/81.1	83.3/81.3	88.8/82.4
	0.02	84.5/82.2	83.0/79.9	80.9/80.0	79.3/75.2	78.1/75.6
	0.03	82.3/80.1	80.4/75.6	77.7/74.3	76.0/72.1	75.2/70.3
	0.04	79.9/74.5	79.4/71.5	75.9/72.3	72.4/70.6	72.6/69.0
	Average	84.0/80.8	82.2/77.1	79.4/76.9	77.8/74.8	78.7/74.3
Total Average		88.4/84.6	83.9/81.0	85.3/80.1	81.2/78.8	82.8/79.0

**Table 4:** Top- 1 classification accuracy of locality preserving project (LPP) and regularized self-representation-locality preserving projects (RSR-LPP) under the proposed method.

Attack Algoritm	Δ	LPP	RSR-LPP
FGSM	0.01	91.5/92.2	96.4/94.0
	0.02	88.2/77.3	94.2/92.3
	0.03	88.1/81.5	91.1/86.0
	0.04	86.0/79.0	89.2/80.9
Deepfool	0.01	88.2/86.9	89.2/86.3
	0.02	82.3/80.9	84.5/82.2
	0.03	81.2/75.3	82.3/80.1
	0.04	79.0/71.8	79.9/74.5

## Data Availability

The data that support the findings of this study are available from the corresponding author upon reasonable request.
